# Isolated Gastrointestinal Presentations in Alpha-Gal Syndrome: A Distinct Clinical Phenotype With High Endoscopic Utilization

**DOI:** 10.1016/j.gastha.2026.101080

**Published:** 2026-07-31

**Authors:** Sameer Rao, Salil Chowdhury, Sima Vossough-Teehan, Amanda A. Rupert, Kaveh Hajifathalian, Ahmed Al-Khazraji

**Affiliations:** 1 Department of Medicine, Rutgers New Jersey Medical School, Newark, New Jersey; 2 Division of Gastroenterology and Hepatology, Rutgers New Jersey Medical School, Newark, New Jersey

Alpha-gal syndrome (AGS) is an immunoglobulin E (IgE)–mediated hypersensitivity reaction to galactose-α-1,3-galactose, a carbohydrate epitope found in non-primate mammalian meat and derived products. Sensitization most commonly follows bites from the lone star tick (*Amblyomma americanum*) in the United States.^1^ Clinically, AGS is characterized by delayed-onset allergic and/or gastrointestinal (GI) manifestations occurring hours after ingestion of red meat, with a subset of patients presenting exclusively with GI symptoms.^2^^,^^3^

AGS is believed to be substantially underdiagnosed, largely due to limited awareness among both healthcare providers and the general population. In a recent Centers for Disease Control and Prevention survey, approximately 42% of healthcare providers reported never having heard of AGS.^4^ Diagnosis relies on a compatible clinical history, including tick exposure and delayed reactions to mammalian meat, with confirmation through detection of alpha-gal–specific IgE. Although thresholds of ≥0.1 kU/L or ≥0.35 kU/L are commonly used to support the diagnosis, seropositivity alone does not equate to clinically significant disease.^5^^,^^6^ Among those who are symptomatic, clinical severity varies widely, ranging from isolated GI symptoms to systemic allergic reactions and anaphylaxis.^2^^,^^7^

To our knowledge, no studies have systematically evaluated baseline clinical differences between AGS patients presenting with isolated GI manifestations and those with non-isolated GI phenotypes at a population level. Furthermore, patterns of endoscopic utilization across these subgroups remain unexplored. In addition, prior investigations examining the relationship between alpha-gal IgE titers and disease severity have yielded mixed results.^2^^,^^8^ To address these gaps, we conducted a large, population-level study to characterize clinical phenotypes, healthcare utilization, and serologic correlates of disease severity in AGS.

We performed a retrospective cohort study using the TriNetX database from January 1, 2010, to November 20, 2025, identifying patients with AGS using International Classification of Diseases, 10th Revision; Current Procedural Terminology; and Logical Observation Identifiers Names and Codes (Supplementary Table 1), with AGS defined by an alpha-gal IgE level ≥0.35 kU/L. Patients were first classified by clinical presentation into an isolated GI group—those with a primary encounter for GI symptoms (diarrhea, nausea, vomiting, or abdominal pain) within 48 hours of a positive alpha-gal IgE test and no documented diagnosis of urticaria, angioedema, or anaphylaxis before or after alpha-gal IgE testing—and a non-isolated GI group, defined by a positive alpha-gal IgE test with documented urticaria, angioedema, or anaphylaxis within 48 hours of testing. Separately, for serologic analyses, all patients with alpha-gal IgE ≥0.35 kU/L were stratified by alpha-gal IgE titers into low-titer (0.35–2 kU/L) and high-titer (>2 kU/L) groups, irrespective of associated allergic symptoms. (Figure). Propensity score matching (1:1) was performed for demographics, allergic comorbidities, and prior GI disorders. Matched cohorts were compared across allergic manifestations, GI diagnoses, and endoscopic procedures. Relative risks (RRs) with 95% confidence intervals were calculated, with *P* < .05 considered statistically significant.

**Figure:**
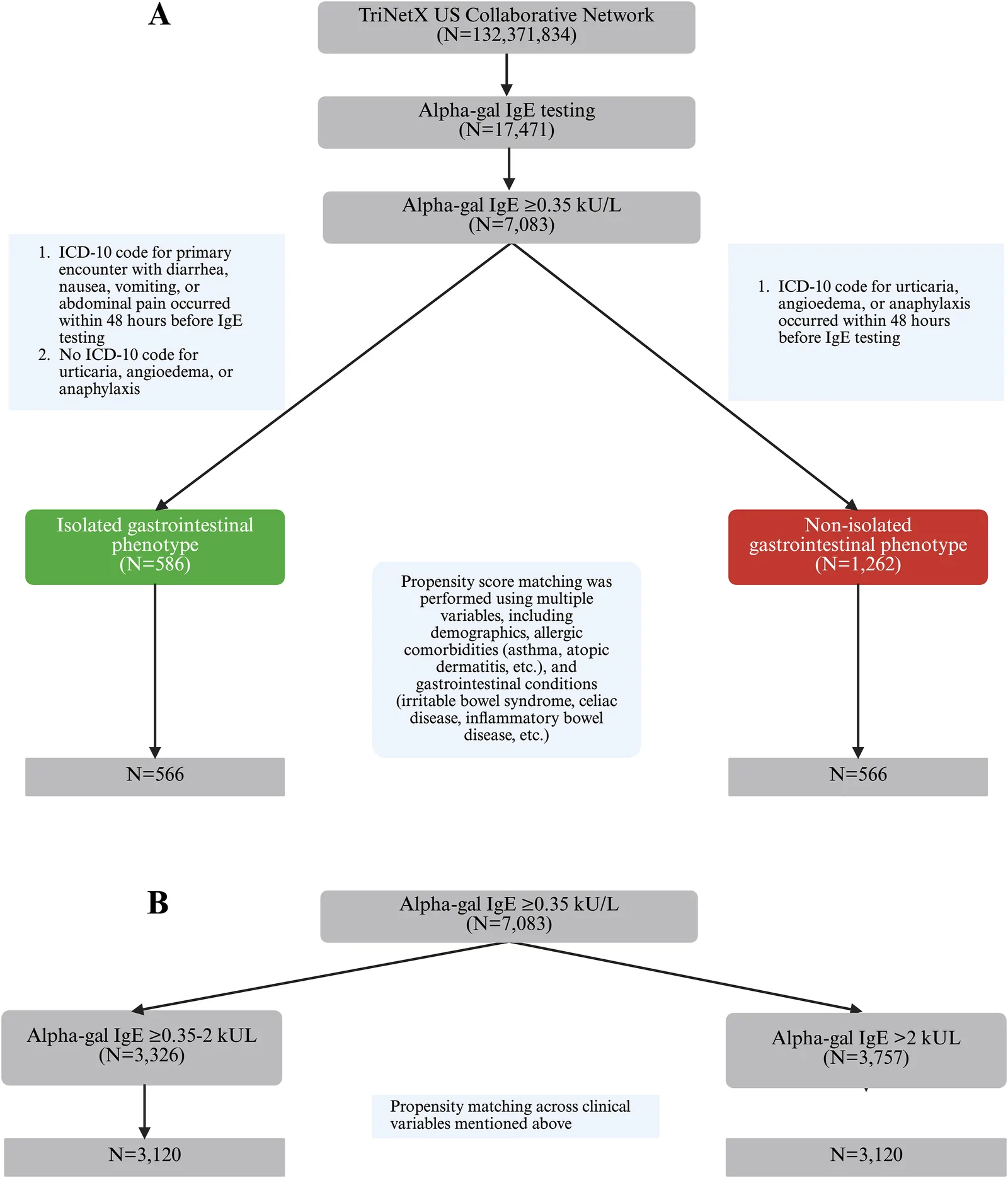
Flowchart depicting cohort selection using the TriNetX US Collaborative Network: (A) isolated and non-isolated GI alpha-gal phenotypes; (B) low-titer and high-titer alpha-gal IgE groups. ICD-10, International Classification of Diseases, 10th Revision.

We identified 586 AGS patients with an isolated GI phenotype and 1262 with non-isolated GI, clinically significant AGS. Patients with isolated GI presentations were younger (47.1 ± 21.8 vs 52 ± 20.4; *P* < .0001) with no differences in gender (female: 48.0% vs 50.6%; *P* = .28) or racial composition between groups. They more frequently carried diagnoses of irritable bowel syndrome (10.92% vs 4.67%; *P* < .0001) and functional dyspepsia (2.56% vs 1.19%; *P* < .0001) (Table). The isolated GI phenotype had higher rates of endoscopy (10.69% vs 2.45%, *P* < .0001) and colonoscopy (4.52% vs 1.47%, *P* < .0001) in 2 years before alpha-gal IgE testing.

**Table:** Baseline Characteristics of the Isolated and Non-Isolated Gastrointestinal Cohorts Before and After Propensity Score Matching

	Characteristic	Isolated GI N (%)	Non-isolated GI N (%)	*P* value^a^
Demographics	Age at testing	Mean 47.1 ± SD 21.8 (N = 586)	Mean 52 ± SD 20.4 (N = 1262)	**< .0001**
		Mean 47.5 ± SD 21.9 (N = 566)	Mean 48.7 ± SD 21.9 (N = 566)	.38
	Female	281 (47.95)	639 (50.63)	.28
		272 (48.05)	279 (49.29)	.68
	White	515 (87.88)	1107 (87.72)	.91
		499 (88.16)	505 (89.22)	.57
	African American	44 (7.51)	64 (5.07)	**.04**
		40 (7.07)	39 (6.89)	.91
	Asian	10 (1.71)	15 (1.19)	.37
		10 (1.77)	10 (1.77)	1
	Unknown race	20 (3.41)	46 (3.64)	.80
		20 (3.53)	16 (2.83)	.50
	Other race	10 (1.71)	22 (1.74)	.95
		10 (1.77)	10 (1.77)	1
Body mass index		Mean 29 ± SD 6.8 (N = 504)	Mean 28.7 ± SD 6.4 (N = 1048)	.32
		Mean 29 ± SD 6.8 (N = 470)	Mean 29.3 ± SD 6.5 (N = 483)	.57
Gastrointestinal diseases	Celiac disease	10 (1.71)	10 (0.79)	.08
		10 (1.77)	10 (1.77)	1
	Crohn’s disease	10 (1.71)	10 (0.79)	.08
		10 (1.77)	10 (1.77)	1
	Fibrosis or cirrhosis of liver	10 (1.71)	10 (0.79)	.08
		10 (1.77)	10 (1.77)	1
	Functional dyspepsia	15 (2.56)	15 (1.19)	**.03**
		12 (2.12)	11 (1.94)	.83
	GERD	267 (45.65)	332 (26.32)	**< .0001**
		239 (42.22)	232 (40.98)	.67
	Irritable bowel syndrome	64 (10.92)	59 (4.67)	**< .0001**
		47 (8.30)	53 (9.36)	.53
	Ulcerative colitis	10 (1.71)	12 (0.95)	.16
		10 (1.77)	10 (1.77)	1
Allergic comorbidities	Allergic rhinitis	156 (26.62)	333 (26.39)	.92
		150 (26.50)	134 (23.67)	.27
	Asthma	91 (15.53)	194 (15.37)	.93
		87 (15.37)	79 (13.96)	.50
	Atopic dermatitis	20 (3.41)	38 (3.01)	.64
		20 (3.53)	18 (3.18)	.74
History of other tick-borne disease	Lyme disease	15 (2.56)	41 (3.25)	.42
		14 (2.47)	10 (1.77)	.41
	Spotted fever	10 (1.71)	14 (1.11)	.29
		10 (1.77)	10 (1.77)	1

For each attribute, upper and lower row represents pre- and post-propensity match data, respectively.

To protect patient privacy, TriNetX rounds all counts ≤10 up to 10; as a result, comparisons in which N is 10 should be interpreted with caution.

GERD, gastroesophageal reflux disease; SD, standard deviation.

a *P* values in bold indicate statistically significant differences.

After 1:1 propensity matching (N = 566 each), isolated GI patients showed greater utilization of upper endoscopy (15.72% vs 2.83%; RR: 5.56; *P* < .0001) and colonoscopy (16.08% vs 4.42%; RR: 3.64; *P* < .0001) over a median follow-up of 14 months with an interquartile range of 24 months. The isolated GI group was less likely to be given or prescribed epinephrine (31.09% vs 39.22%; RR: 0.79; *P* = .004).

Among serologic subgroups, 3326 patients had low alpha-gal IgE titers and 3757 had high titers. Low-titer patients were younger (51.9 ± 20.4 vs 55.6 ± 18.7; *P* < .0001) and more often female (53.76% vs 46.50%; *P* < .0001) with no differences in racial composition (Supplementary Table 2).

After matching (N = 3120 each), the low-titer group had lower rates of anaphylaxis compared with the high-titer group (3.40% vs 5.35%; RR: 0.63; *P* = .0002) over a median follow-up of 17.8 months with an interquartile range of 30.5 months.

In our study, patients with AGS presenting with an isolated GI phenotype were younger and more frequently assigned diagnoses of irritable bowel syndrome and functional dyspepsia prior to alpha-gal IgE testing. This pattern suggests possible diagnostic delay and misattribution of symptoms to disorders of gut–brain interaction before recognition of an allergic etiology.^7^ The isolated GI phenotype is increasingly recognized as a frequent presentation of AGS but remains substantially underdiagnosed due to nonspecific symptoms and limited awareness among healthcare providers.

Patients with the isolated GI phenotype also demonstrated significantly higher endoscopic utilization both before and after alpha-gal IgE testing. This finding likely reflects the use of endoscopic evaluation to exclude structural, inflammatory, or malignant GI disease before considering AGS as the underlying diagnosis for the GI symptoms. In patients with alpha-gal IgE seropositivity and a clinically compatible history of delayed symptoms following mammalian meat ingestion, the role of endoscopy may be best reserved for those presenting with alarm features suggestive of structural GI pathology.

To explore disease severity, we used the presence of anaphylaxis as a clinical marker and stratified AGS patients by alpha-gal IgE titers into low- and high-titer groups. A cutoff of 2 kU/L was selected based on prior evidence identifying this threshold as optimal for predicting a positive oral food challenge in patients with AGS.^9^ Patients in the low-titer group (alpha-gal IgE 0.35–2 kU/L) exhibited lower rates of anaphylaxis compared with those in the high-titer group (alpha-gal IgE >2 kU/L). It is possible that lower alpha-gal IgE levels capture individuals with serologic sensitization in the absence of overt clinical manifestations.^10^ Additionally, higher levels may be associated with an increased risk of anaphylaxis due to a greater burden of antigen-specific IgE available for cross-linking and subsequent systemic mast cell activation.^8^ Both scenarios may coexist across the clinical spectrum of AGS; however, further studies with granular clinical data are needed to better characterize these underlying mechanisms.

This study has limitations inherent to retrospective database analyses. AGS phenotype classification (isolated vs non-isolated GI) was based on diagnostic codes; this approach may not fully capture clinical nuance, and some degree of misclassification is possible due to limited clinical granularity. Although propensity score matching was performed, residual unmeasured confounding should be considered when interpreting these findings. Lastly, clinical database studies are limited in capturing anaphylaxis, as some patients may self-treat at home without diagnostic coding.

In conclusion, the isolated GI phenotype of AGS represents a younger subgroup more frequently associated with coexisting diagnoses of disorders of gut–brain interaction and higher endoscopic utilization, highlighting challenges in clinical recognition of this presentation. Additionally, higher alpha-gal IgE levels were associated with increased risk of anaphylaxis, supporting a potential relationship between serologic burden and disease severity.

## Supplementary Materials

Supplementary Tables

Extended PDF
