# Supplementary material for: Isolated Gastrointestinal Presentations in Alpha-Gal Syndrome: A Distinct Clinical Phenotype With High Endoscopic Utilization
Source: Gastro Hep Adv. 2026 Jul 31;5(11):101080. doi: 10.1016/j.gastha.2026.101080 (PMC13524450; doi:10.1016/j.gastha.2026.101080)
Supplement: Supplementary file 1 [file mmc1.pdf]

**Supplementary Table 1** - Attributes and corresponding ICD-10 (international classification of diseases, 10th revision), CPT (current procedural terminology), LOINC (logical observation identifiers names and codes), or Rxnorm codes used in the study

| Attribute | ICD-10/CPT/LOINC/RxNorm |
|---|---|
| | |
| **Abdominal pain** | R10.9 |
| **Allergic rhinitis** | J30 |
| **Anaphylaxis** | T78.2 |
| **Angioneurotic edema** | T78.3 |
| **Asthma** | J45 |
| **Atopic dermatitis** | L20 |
| **Celiac disease** | K90.0 |
| **Colonoscopy** | 45378, 45380, 45385 |
| **Crohn's disease** | K50 |
| **Diarrhea** | R19.7 |
| **Epinephrine** | 3992 |
| **Esophagogastroduodenoscopy** | 43235, 43239 |
| **Functional dyspepsia** | K30 |
| **Galactose-alpha-1,3-galactose IgE Ab** | 73837-7 |
| **Gastroesophageal reflux disease** | K21 |
| **Irritable bowel syndrome** | K58 |
| **Liver cirrhosis** | K74.60 |
| **Lyme disease** | A69.2 |
| **Nausea and vomiting** | R11 |
| **Spotted fever** | A77 |
| **Ulcerative colitis** | K51 |
| **Urticaria** | L50 |

**Supplementary Table 2**: Baseline characteristics of the low and high alpha-gal IgE cohorts before and after propensity score matching

| | Characteristic | Low alpha-gal IgE titers N (%) | High alpha-gal IgE titers N (%) | P-value |
|---|---|---|---|---|
| | | | | |
| | Age at testing | Mean 51.9 ± SD 20.4 | Mean 55.6 ± SD 18.7 | **<.0001** |

| | | | | |
|---|---|---|---|---|
| Demographics | | (N=3,326) | (N=3,757) | |
| | | Mean 53.6 ± SD 19.6 (N=3,120) | Mean 53.6 ± SD 19.2 (N=3,120) | .90 |
| | Female | 1,788 (53.76) | 1,747 (46.50) | **<.0001** |
| | | 1,603 (51.38) | 1,620 (51.92) | .67 |
| | White | 2,981 (89.63) | 3,374 (89.81) | .80 |
| | | 2,794 (89.55) | 2,816 (90.26) | .36 |
| | African American | 135 (4.06) | 155 (4.13) | .89 |
| | | 130 (4.17) | 122 (3.91) | .61 |
| | Asian | 24 (0.72) | 29 (0.77) | .81 |
| | | 24 (0.77) | 22 (0.70) | .77 |
| | Unknown race | 124 (3.73) | 139 (3.70) | .95 |
| | | 117 (3.75) | 110 (3.53) | .64 |
| | Other race | 44 (1.32) | 45 (1.20) | .64 |
| | | 39 (1.25) | 36 (1.15) | .72 |
| **Body mass index** | | Mean 28.6 ± SD 6.7 (N=2,920) | Mean 28.7 ± SD 6.3 (N=3,277) | .84 |
| | | Mean 28.7 ± SD 6.6 (N=2,733) | Mean 28.7 ± SD 6.5 (N=2,719) | .66 |
| **Gastrointestinal diseases** | Celiac disease | 31 (0.93) | 30 (0.80) | .54 |
| | | 28 (0.90) | 28 (0.90) | 1 |
| | Crohn's disease | 23 (0.69) | 28 (0.74) | .79 |
| | | 23 (0.74) | 23 (0.74) | 1 |
| | Fibrosis or cirrhosis of liver | 24 (0.71) | 38 (1.02) | .15 |
| | | 24 (0.76) | 24 (0.76) | 1 |
| | Functional dyspepsia | 75 (2.25) | 69 (1.84) | .21 |
| | | 63 (2.02) | 63 (2.02) | 1 |
| | GERD | 1,186 (35.66) | 1,251 (33.30) | **.03** |
| | | 1,084 (34.97) | 1,077 (34.52) | .71 |
| | Irritable bowel syndrome | 291 (8.75) | 278 (7.40) | **.04** |
| | | 250 (8.01) | 243 (7.79) | .74 |
| | Ulcerative colitis | 38 (1.14) | 39 (1.04) | .67 |
| | | 34 (1.09) | 34 (1.09) | 1 |
| **Allergic co-morbidities** | Allergic rhinitis | 983 (29.56) | 1,005 (26.75) | **.009** |
| | | 895 (28.69) | 879 (28.17) | .65 |
| | Asthma | 585 (17.59) | 623 (16.58) | .26 |
| | | 543 (17.40) | 534 (17.11) | .74 |
| | Atopic dermatitis | 116 (3.49) | 118 (3.14) | .41 |
| | | 103 (3.30) | 100 (3.20) | .83 |
| **History of other tick-borne disease** | Lyme disease | 154 (4.63) | 162 (4.31) | .52 |
| | | 148 (4.74) | 126 (4.04) | .17 |
| | Spotted fever | 59 (1.77) | 72 (1.92) | .66 |
| | | 59 (1.89) | 42 (1.35) | .09 |
| | | | | |
| For each attribute, upper and lower row represents pre- and post-propensity match data respectively | | | | |

To protect patient privacy, TriNetX rounds all counts ≤10 up to 10; as a result, comparisons in which N is 10 should be interpreted with caution

**Abbreviations used**: GERD – gastroesophageal reflux disease, GI – gastrointestinal, SD – standard deviation
