# Supplementary material for: Isolated Gastrointestinal Presentations in Alpha-Gal Syndrome: A Distinct Clinical Phenotype With High Endoscopic Utilization
Source: Gastro Hep Adv. 2026 Jul 31;5(11):101080. doi: 10.1016/j.gastha.2026.101080 (PMC13524450; doi:10.1016/j.gastha.2026.101080)
Supplement: Supplementary file 2 [file mmc2.pdf]

Gastro Hep Advances 2026;5:101080

RESEARCH LETTERS

Isolated Gastrointestinal Presentations in Alpha-Gal Syndrome: A Distinct Clinical Phenotype With High Endoscopic Utilization

Alpha-gal syndrome (AGS) is an immunoglobulin E (IgE)–mediated hypersensitivity reaction to galactose-α-1,3-galactose, a carbohydrate epitope found in non-primate mammalian meat and derived products. Sensitization most commonly follows bites from the lone star tick (*Amblyomma americanum*) in the United States.[1] Clinically, AGS is characterized by delayed-onset allergic and/or gastrointestinal (GI) manifestations occurring hours after ingestion of red meat, with a subset of patients presenting exclusively with GI symptoms.[2,3]

AGS is believed to be substantially underdiagnosed, largely due to limited awareness among both healthcare providers and the general population. In a recent Centers for Disease Control and Prevention survey, approximately 42% of healthcare providers reported never having heard of AGS.[4] Diagnosis relies on a compatible clinical history, including tick exposure and delayed reactions to mammalian meat, with confirmation through detection of alpha-gal–specific IgE. Although thresholds of ≥0.1 kU/L or ≥0.35 kU/L are commonly used to support the diagnosis, seropositivity alone does not equate to clinically significant disease.[5,6] Among those who are symptomatic, clinical severity varies widely, ranging from isolated GI symptoms to systemic allergic reactions and anaphylaxis.[2,7]

To our knowledge, no studies have systematically evaluated baseline clinical differences between AGS patients presenting with isolated GI manifestations and those with non-isolated GI phenotypes at a population level. Furthermore, patterns of endoscopic utilization across these subgroups remain unexplored. In addition, prior investigations examining the relationship between alpha-gal IgE titers and disease severity have yielded mixed results.[2,8] To address these gaps, we conducted a large, population-level study to characterize clinical phenotypes, healthcare utilization, and serologic correlates of disease severity in AGS.

We performed a retrospective cohort study using the TriNetX database from January 1, 2010, to November 20, 2025, identifying patients with AGS using International Classification of Diseases, 10th Revision; Current Procedural Terminology; and Logical Observation Identifiers Names and Codes (Supplementary Table 1), with AGS defined by an alpha-gal IgE level ≥0.35 kU/L. Patients were first classified by clinical presentation into an isolated GI group—those with a primary encounter for GI symptoms (diarrhea, nausea, vomiting, or abdominal pain) within 48 hours of a positive alpha-gal IgE test and no documented diagnosis of urticaria, angioedema, or anaphylaxis before or after alpha-gal IgE testing—and a non-isolated GI group, defined by a positive alpha-gal IgE test with documented urticaria, angioedema, or anaphylaxis within 48 hours of testing. Separately, for serologic analyses, all patients with alpha-gal IgE ≥0.35 kU/L were stratified by alpha-gal IgE titers into low-titer (0.35–2 kU/L) and high-titer (>2 kU/L) groups, irrespective of associated allergic symptoms. (Figure). Propensity score matching (1:1) was performed for demographics, allergic comorbidities, and prior GI disorders. Matched cohorts were compared across allergic manifestations, GI diagnoses, and endoscopic procedures. Relative risks (RRs) with 95% confidence intervals were calculated, with $P < .05$ considered statistically significant.

We identified 586 AGS patients with an isolated GI phenotype and 1262 with non-isolated GI, clinically significant AGS. Patients with isolated GI presentations were younger (47.1 ± 21.8 vs 52 ± 20.4; $P < .0001$) with no differences in gender (female: 48.0% vs 50.6%; $P = .28$) or racial composition between groups. They more frequently carried diagnoses of irritable bowel syndrome (10.92% vs 4.67%; $P < .0001$) and functional dyspepsia (2.56% vs 1.19%; $P < .0001$) (Table). The isolated GI phenotype had higher rates of endoscopy (10.69% vs 2.45%, $P < .0001$) and colonoscopy (4.52% vs 1.47%, $P < .0001$) in 2 years before alpha-gal IgE testing.

After 1:1 propensity matching (N = 566 each), isolated GI patients showed greater utilization of upper endoscopy (15.72% vs 2.83%; RR: 5.56; $P < .0001$) and colonoscopy (16.08% vs 4.42%; RR: 3.64; $P < .0001$) over a median follow-up of 14 months with an interquartile range of 24 months. The isolated GI group was less likely to be given or prescribed epinephrine (31.09% vs 39.22%; RR: 0.79; $P = .004$).

Among serologic subgroups, 3326 patients had low alpha-gal IgE titers and 3757 had high titers. Low-titer patients were younger (51.9 ± 20.4 vs 55.6 ± 18.7; $P < .0001$) and more often female (53.76% vs 46.50%; $P < .0001$) with no differences in racial composition (Supplementary Table 2).

After matching (N = 3120 each), the low-titer group had lower rates of anaphylaxis compared with the high-titer group (3.40% vs 5.35%; RR: 0.63; $P = .0002$) over a median follow-up of 17.8 months with an interquartile range of 30.5 months.

In our study, patients with AGS presenting with an isolated GI phenotype were younger and more frequently assigned diagnoses of irritable bowel syndrome and functional dyspepsia prior to alpha-gal IgE testing. This pattern suggests possible diagnostic delay and misattribution of symptoms to disorders of gut–brain interaction before recognition of an allergic etiology.[7] The isolated GI phenotype is increasingly recognized as a frequent presentation of AGS but remains substantially underdiagnosed due to nonspecific symptoms

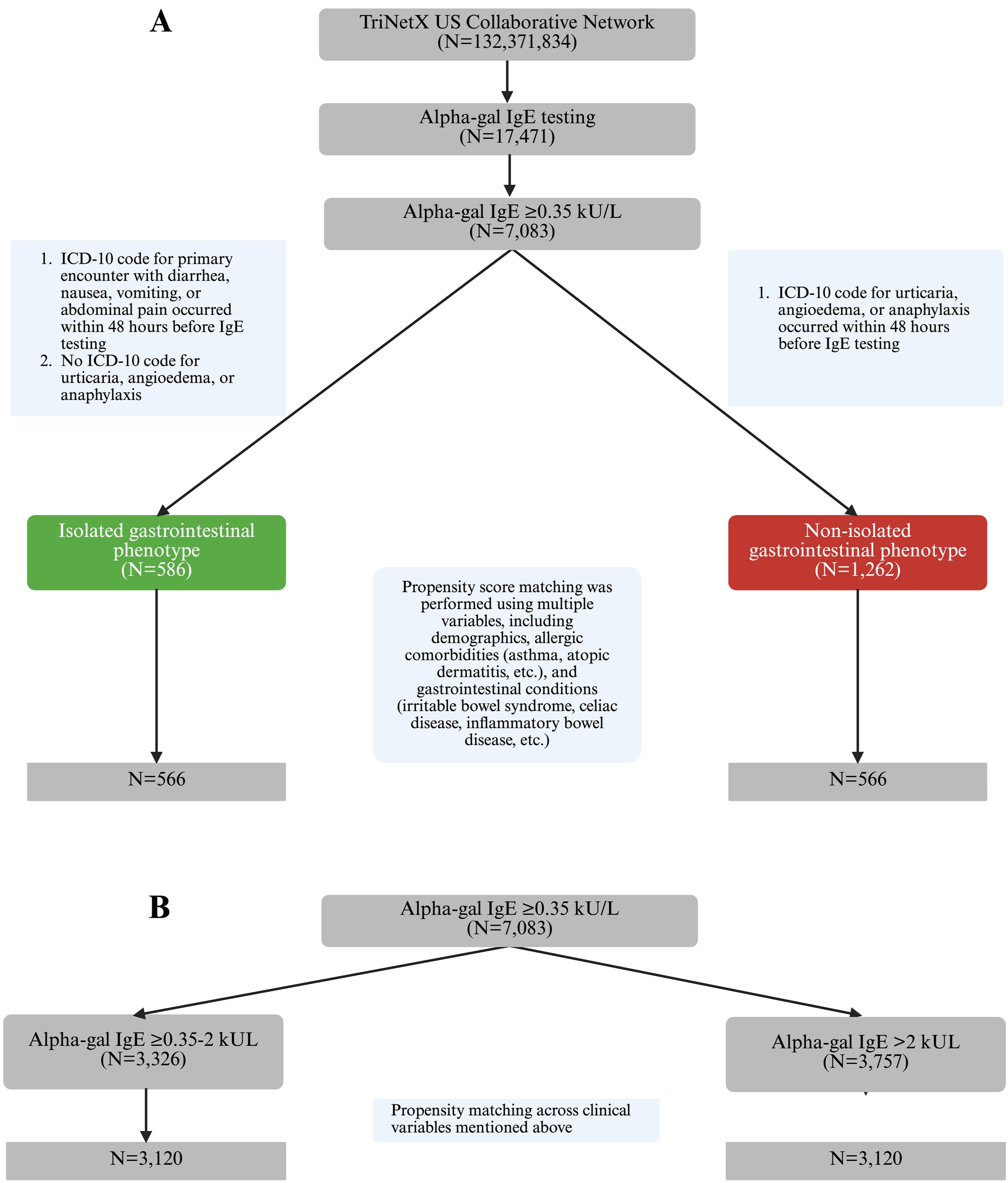

Figure. Flowchart depicting cohort selection using the TriNetX US Collaborative Network: (A) isolated and non-isolated GI alpha-gal phenotypes; (B) low-titer and high-titer alpha-gal IgE groups. ICD-10, International Classification of Diseases, 10th Revision.

Table. Baseline Characteristics of the Isolated and Non-Isolated Gastrointestinal Cohorts Before and After Propensity Score Matching

	Characteristic	Isolated GI N (%)	Non-isolated GI N (%)	*P* value[a]
Demographics	Age at testing	Mean 47.1 ± SD 21.8 (N = 586)	Mean 52 ± SD 20.4 (N = 1262)	**< .0001**
		Mean 47.5 ± SD 21.9 (N = 566)	Mean 48.7 ± SD 21.9 (N = 566)	.38
	Female	281 (47.95)	639 (50.63)	.28
		272 (48.05)	279 (49.29)	.68
	White	515 (87.88)	1107 (87.72)	.91
		499 (88.16)	505 (89.22)	.57
	African American	44 (7.51)	64 (5.07)	**.04**
		40 (7.07)	39 (6.89)	.91
	Asian	10 (1.71)	15 (1.19)	.37
		10 (1.77)	10 (1.77)	1
	Unknown race	20 (3.41)	46 (3.64)	.80
		20 (3.53)	16 (2.83)	.50
	Other race	10 (1.71)	22 (1.74)	.95
		10 (1.77)	10 (1.77)	1
Body mass index		Mean 29 ± SD 6.8 (N = 504)	Mean 28.7 ± SD 6.4 (N = 1048)	.32
		Mean 29 ± SD 6.8 (N = 470)	Mean 29.3 ± SD 6.5 (N = 483)	.57
Gastrointestinal diseases	Celiac disease	10 (1.71)	10 (0.79)	.08
		10 (1.77)	10 (1.77)	1
	Crohn's disease	10 (1.71)	10 (0.79)	.08
		10 (1.77)	10 (1.77)	1
	Fibrosis or cirrhosis of liver	10 (1.71)	10 (0.79)	.08
		10 (1.77)	10 (1.77)	1
	Functional dyspepsia	15 (2.56)	15 (1.19)	**.03**
		12 (2.12)	11 (1.94)	.83
	GERD	267 (45.65)	332 (26.32)	**< .0001**
		239 (42.22)	232 (40.98)	.67
	Irritable bowel syndrome	64 (10.92)	59 (4.67)	**< .0001**
		47 (8.30)	53 (9.36)	.53
	Ulcerative colitis	10 (1.71)	12 (0.95)	.16
		10 (1.77)	10 (1.77)	1
Allergic comorbidities	Allergic rhinitis	156 (26.62)	333 (26.39)	.92
		150 (26.50)	134 (23.67)	.27
	Asthma	91 (15.53)	194 (15.37)	.93
		87 (15.37)	79 (13.96)	.50
	Atopic dermatitis	20 (3.41)	38 (3.01)	.64
		20 (3.53)	18 (3.18)	.74
History of other tick-borne disease	Lyme disease	15 (2.56)	41 (3.25)	.42
		14 (2.47)	10 (1.77)	.41
	Spotted fever	10 (1.71)	14 (1.11)	.29
		10 (1.77)	10 (1.77)	1

For each attribute, upper and lower row represents pre- and post-propensity match data, respectively.
To protect patient privacy, TriNetX rounds all counts ≤10 up to 10; as a result, comparisons in which N is 10 should be interpreted with caution.
GERD, gastroesophageal reflux disease; SD, standard deviation.
[a]*P* values in bold indicate statistically significant differences.

and limited awareness among health-care providers.

Patients with the isolated GI phenotype also demonstrated significantly higher endoscopic utilization both before and after alpha-gal IgE testing. This finding likely reflects the use of endoscopic evaluation to exclude structural, inflammatory, or malignant GI disease before considering AGS as the underlying diagnosis for the GI symptoms. In patients with alpha-gal IgE seropositivity and a clinically compatible history of delayed symptoms following mammalian meat ingestion, the role of endoscopy may be best reserved for those presenting with alarm features suggestive of structural GI pathology.

To explore disease severity, we used the presence of anaphylaxis as a clinical marker and stratified AGS patients by alpha-gal IgE titers into low- and high-titer groups. A cutoff of 2 kU/L was selected based on prior evidence identifying this threshold as optimal for predicting a positive oral food challenge in patients with AGS.[9] Patients in the low-titer group (alpha-gal IgE 0.35–2 kU/L) exhibited lower rates of anaphylaxis compared with those in the high-titer group (alpha-gal IgE >2 kU/L). It is possible that lower alpha-gal IgE levels capture individuals with serologic sensitization in the absence of overt clinical manifestations.[10] Additionally, higher levels may be associated with an increased risk of anaphylaxis due to a greater burden of antigen-specific IgE available for cross-linking and subsequent systemic mast cell activation.[8] Both scenarios may coexist across the clinical spectrum of AGS; however, further studies with granular clinical data are needed to better characterize these underlying mechanisms.

This study has limitations inherent to retrospective database analyses. AGS phenotype classification (isolated vs non-isolated GI) was based on diagnostic codes; this approach may not fully capture clinical nuance, and some degree of misclassification is possible due to limited clinical granularity. Although propensity score matching was performed, residual unmeasured confounding should be considered when interpreting these findings. Lastly, clinical database studies are limited in capturing anaphylaxis, as some patients may self-treat at home without diagnostic coding.

In conclusion, the isolated GI phenotype of AGS represents a younger subgroup more frequently associated with coexisting diagnoses of disorders of gut–brain interaction and higher endoscopic utilization, highlighting challenges in clinical recognition of this presentation. Additionally, higher alpha-gal IgE levels were associated with increased risk of anaphylaxis, supporting a potential relationship between serologic burden and disease severity.

SAMEER RAO[1]
SALIL CHOWDHURY[2]
SIMA VOSSOUGH-TEEHAN[2]
AMANDA A. RUPERT[2]
KAVEH HAJIFATHALIAN[2]
AHMED AL-KHAZRAJI[2]
[1]Department of Medicine, Rutgers New Jersey Medical School, Newark, New Jersey
[2]Division of Gastroenterology and Hepatology, Rutgers New Jersey Medical School, Newark, New Jersey

Correspondence:
Address correspondence to: Sameer Rao, MBBS, Department of Medicine, Rutgers New Jersey Medical School, Newark, NJ. e-mail: sr2193@njms.rutgers.edu.

Supplementary Materials

Material associated with this article can be found, in the online version, at https://doi.org/10.1016/j.gastha.2026.101080.

References

1. Commins SP, et al. J Allergy Clin Immunol 2009;123(2):426–433.
2. Lesmana E, et al. Clin Gastroenterol Hepatol 2025;23(1):69–78.
3. Richards NE, et al. South Med J 2021;114(3):169–173.
4. Carpenter A, et al. MMWR Morb Mortal Wkly Rep 2023;72(30):809–814.
5. McGill SK, et al. Clin Gastroenterol Hepatol 2023;21(4):891–896.
6. Germán-Sánchez A, et al. Allergy Asthma Clin Immunol 2023;19(1):102.
7. Croglio MP, et al. Gastroenterology 2021;160(6):2178.
8. Kiewiet MBG, et al. J Allergy Clin Immunol Pract 2020;8(6):2027–2034.e2.
9. Mabelane T, et al. Pediatr Allergy Immunol 2018;29(8):841–849.
10. Fischer J, et al. Allergy 2017;72(10):1540–1547.

Abbreviations used in this paper: **AGS, alpha-gal syndrome; GI, gastrointestinal; Ig, immunoglobulin; RR, relative risk**

Most current article

© 2026 The Author(s). Published by Elsevier Inc. on behalf of American Gastroenterological Association Institute. This is an open access article under the CC BY license (http://creativecommons.org/licenses/by/4.0/).
2772-5723
https://doi.org/10.1016/j.gastha.2026.101080

Received February 24, 2026. Accepted July 27, 2026.

Authors' Contributions:
Sameer Rao: Conceptualization, study design, data extraction and analysis, interpretation of results, and co-lead in original draft writing. Salil Chowdhury: Conceptualization, interpretation of results, and co-lead in original draft writing. Sima Vossough-Teehan: Conceptualization, interpretation of results, and manuscript review and editing. Amanda A. Rupert: Supervision, conceptualization, and co-lead in manuscript review and editing. Kaveh Hajifathalian: Supervision, data interpretation, and manuscript review and editing. Ahmed Al-Khazraji: Supervision, conceptualization, study design, project oversight, and co-lead in manuscript review and editing.

Conflicts of Interest:
The authors disclose no conflicts. An abstract based was presented as a poster at Digestive Disease Week 2026 in Chicago, IL, USA.

Funding:
The authors report no funding.

Ethical Statement:
Not required, as this was a database study using deidentified data.

Data Transparency Statement:
Data for this study were obtained from the TriNetX Research Network under institutional license and are not publicly available due to data use agreements.

Reporting Guidelines:
This study followed the Strengthening the Reporting of Observational Studies in Epidemiology (STROBE) reporting guideline.

Gastro Hep Advances, Volume *5*

Supplemental information

Isolated Gastrointestinal Presentations in Alpha-Gal Syndrome: A Distinct Clinical Phenotype With High Endoscopic Utilization

Sameer Rao, Salil Chowdhury, Sima Vossough-Teehan, Amanda A. Rupert, Kaveh Hajifathalian, and Ahmed Al-Khazraji

Supplementary Table 1 - Attributes and corresponding ICD-10 (international classification of diseases, 10th revision), CPT (current procedural terminology), LOINC (logical observation identifiers names and codes), or Rxnorm codes used in the study

Attribute	ICD-10/CPT/LOINC/RxNorm
**Abdominal pain**	R10.9
**Allergic rhinitis**	J30
**Anaphylaxis**	T78.2
**Angioneurotic edema**	T78.3
**Asthma**	J45
**Atopic dermatitis**	L20
**Celiac disease**	K90.0
**Colonoscopy**	45378, 45380, 45385
**Crohn's disease**	K50
**Diarrhea**	R19.7
**Epinephrine**	3992
**Esophagogastroduodenoscopy**	43235, 43239
**Functional dyspepsia**	K30
**Galactose-alpha-1,3-galactose IgE Ab**	73837-7
**Gastroesophageal reflux disease**	K21
**Irritable bowel syndrome**	K58
**Liver cirrhosis**	K74.60
**Lyme disease**	A69.2
**Nausea and vomiting**	R11
**Spotted fever**	A77
**Ulcerative colitis**	K51
**Urticaria**	L50

Supplementary Table 2: Baseline characteristics of the low and high alpha-gal IgE cohorts before and after propensity score matching

	Characteristic	Low alpha-gal IgE titers N (%)	High alpha-gal IgE titers N (%)	P-value
	Age at testing	Mean 51.9 ± SD 20.4	Mean 55.6 ± SD 18.7	**<.0001**

Demographics		(N=3,326)	(N=3,757)	
		Mean 53.6 ± SD 19.6 (N=3,120)	Mean 53.6 ± SD 19.2 (N=3,120)	.90
	Female	1,788 (53.76)	1,747 (46.50)	**<.0001**
		1,603 (51.38)	1,620 (51.92)	.67
	White	2,981 (89.63)	3,374 (89.81)	.80
		2,794 (89.55)	2,816 (90.26)	.36
	African American	135 (4.06)	155 (4.13)	.89
		130 (4.17)	122 (3.91)	.61
	Asian	24 (0.72)	29 (0.77)	.81
		24 (0.77)	22 (0.70)	.77
	Unknown race	124 (3.73)	139 (3.70)	.95
		117 (3.75)	110 (3.53)	.64
	Other race	44 (1.32)	45 (1.20)	.64
		39 (1.25)	36 (1.15)	.72
**Body mass index**		Mean 28.6 ± SD 6.7 (N=2,920)	Mean 28.7 ± SD 6.3 (N=3,277)	.84
		Mean 28.7 ± SD 6.6 (N=2,733)	Mean 28.7 ± SD 6.5 (N=2,719)	.66
**Gastrointestinal diseases**	Celiac disease	31 (0.93)	30 (0.80)	.54
		28 (0.90)	28 (0.90)	1
	Crohn's disease	23 (0.69)	28 (0.74)	.79
		23 (0.74)	23 (0.74)	1
	Fibrosis or cirrhosis of liver	24 (0.71)	38 (1.02)	.15
		24 (0.76)	24 (0.76)	1
	Functional dyspepsia	75 (2.25)	69 (1.84)	.21
		63 (2.02)	63 (2.02)	1
	GERD	1,186 (35.66)	1,251 (33.30)	**.03**
		1,084 (34.97)	1,077 (34.52)	.71
	Irritable bowel syndrome	291 (8.75)	278 (7.40)	**.04**
		250 (8.01)	243 (7.79)	.74
	Ulcerative colitis	38 (1.14)	39 (1.04)	.67
		34 (1.09)	34 (1.09)	1
**Allergic co-morbidities**	Allergic rhinitis	983 (29.56)	1,005 (26.75)	**.009**
		895 (28.69)	879 (28.17)	.65
	Asthma	585 (17.59)	623 (16.58)	.26
		543 (17.40)	534 (17.11)	.74
	Atopic dermatitis	116 (3.49)	118 (3.14)	.41
		103 (3.30)	100 (3.20)	.83
**History of other tick-borne disease**	Lyme disease	154 (4.63)	162 (4.31)	.52
		148 (4.74)	126 (4.04)	.17
	Spotted fever	59 (1.77)	72 (1.92)	.66
		59 (1.89)	42 (1.35)	.09
For each attribute, upper and lower row represents pre- and post-propensity match data respectively				

To protect patient privacy, TriNetX rounds all counts ≤10 up to 10; as a result, comparisons in which N is 10 should be interpreted with caution

Abbreviations used: GERD – gastroesophageal reflux disease, GI – gastrointestinal, SD – standard deviation
